# Clinical Analysis of Castleman's Disease of the Lacrimal Gland

**DOI:** 10.1155/2020/3718305

**Published:** 2020-11-23

**Authors:** Dongping Li, Dongrun Tang, Fengyuan Sun

**Affiliations:** ^1^Eye Institute and School of Optometry, Tianjin Medical University Eye Hospital, Tianjin 300384, China; ^2^Tianjin International Joint Research and Development Centre of Ophthalmology and Vision Science, Tianjin 300384, China

## Abstract

**Objective:**

To explore the clinical manifestations, imaging characteristics, and pathological characteristics of Castleman's disease of the lacrimal gland, enhance the knowledge of the disease, and improve the level of its diagnosis and treatment.

**Methods:**

In the retrospective study, the data of 5 patients diagnosed with Castleman's disease of the lacrimal gland in Tianjin Medical University Eye Hospital from 2014 to 2018 were analyzed, and the relevant literature was reviewed.

**Results:**

All the 5 patients were confirmed by pathological examination. Clinical manifestations were characterized by mass occupying lesions in the lacrimal gland area, without obvious pain, accompanied by eyelid swelling and ptosis, as well as space-occupying symptoms. Imaging examination showed that there was a soft tissue mass in the enlarged lacrimal gland area, and the mass was rich in blood flows while showing no obvious specificity, which could invade the surrounding muscles. All patients underwent surgical resection. Pathological results showed that 1 case was of the hyaline-vascular type, 3 cases were of the plasma cell type, and 1 case showed malignant transformation to plasma cell tumor.

**Conclusion:**

Castleman's disease of the lacrimal gland is a rare orbital lymphoproliferative disease lacking specificity in clinical manifestations and imaging examination. As there are difficulties in differentiating the disease from orbital inflammatory pseudotumor and orbital lymphoma, its diagnosis still depends on pathological examination. The disease is mainly treated with surgical resection, and the pathological type is determined postoperatively.

## 1. Introduction

Castleman's disease (CD) is a rare chronic lymphoproliferative disease in the clinic, usually involving the mediastinum, neck, retroperitoneum, axilla, groin, and other regions rich in lymph nodes [1]. Orbital involvement is rarely seen. It was first reported as a mediastinal mass similar to a thymoma by Benjamin Castleman in 1956 [2], and the first case involving the eye was reported by Gittinger in 1989 [3]. In recent years, scholars have reported CD cases involving the whole body, as well as the eyes. CD mainly involves lymph nodes, and extranodal tissue involvement is noted occasionally. Pathologically, it includes the hyaline-vascular type, plasma cell type, and mixed type. The lacrimal gland is an orbital autoimmune organ, which is a common site of orbital lymphoproliferative diseases. The author received and treated 5 cases of CD involving the lacrimal gland from 2014 to 2018 and analyzed their clinical manifestations, imaging characteristics, and pathological features in the combination of the relevant literature, aiming to enhance the knowledge of ophthalmologists on the disease and improve the diagnosis and treatment level.

## 2. Clinical Data

### 2.1. Study Population

The study was approved by the Foundation Institutional Review Board of Tianjin Medical University Eye Hospital and adhered to HIPAA regulations and the principles of the *Declaration of Helsinki*. Five patients included in this study were pathologically diagnosed as CD from January 2014 to September 2018 at Tianjin Medical University Eye Hospital.

### 2.2. Study Methods

Clinical data of the patients were collected, including (**A**) general data (gender, age, and eye type), (**B)** clinical manifestations (vision, disease course, lesion site, and inflammation), (**C)** imaging data, (**D)** pathological data, and (**E**) treatment and prognosis.

## 3. Results

### 3.1. Characteristics of the Study Population and Clinical Manifestations

Among the 5 patients, there were 1 male and 4 female, with an average age of 60.1 years (44 –76 y). The lacrimal gland was involved in all cases, unilateral in 3 cases and bilateral in 2 cases. The disease was onset 2 months to 60 months ago, with the mass occupying the lacrimal gland area without obvious pain, accompanied by eyelid swelling, ptosis of the upper eyelid, and partial invasion of the eye muscle leading to restricted eye movement and diplopia in all cases. One patient with recurrence presented an obvious malignant trend. One patient was accompanied by sublingual gland lobular enlargement, one patient showed CMV(+), HSV-1(+), and VZV(+) in the blood test, and three patients had no systemic symptoms. All 5 patients were treated with surgical resection. Except for Case 4, all the other patients received postoperative hormone therapy, with effects to different extends. Pathology revealed 1 case of hyaline-vascular type, 3 cases of plasma cell type, and 1 case of malignant transformation to the plasma cell tumor. During the 2–5 years of follow-up, 3 patients had no recurrence while the other 2 patients had. Among them, 1 patient was presented with malignant transformation and was further treated with chemotherapy in the tumor hospital. Another patient developed a kind of IgG 4-related eye disease and improved with hormone therapy. Two patients died, Case 1 due to cardiovascular accident and Case 4 due to progressive disease (Table 1).

### 3.2. Pathological Features (Table 2)

#### 3.2.1. Introduction of Typical Cases

**Figure 1:**
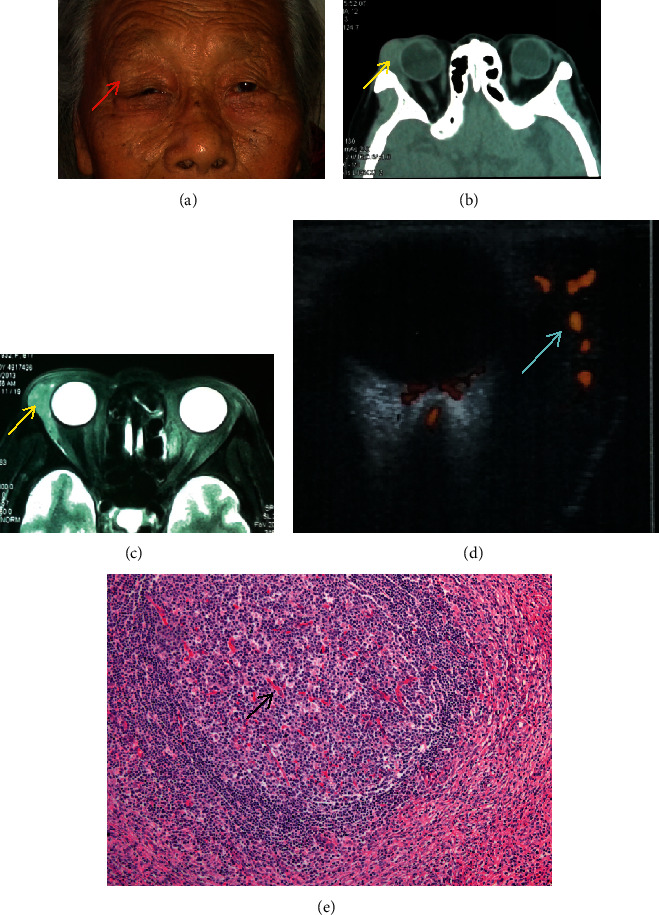
(a), (b), (c). Preoperative appearance of the right eye. CT and MRI showed a swollen mass in the lacrimal gland area (red and yellow arrows). (d) Color ultrasonography showed a mass in the lacrimal gland area of the right eye with abundant blood flow (blue arrow). (e) Small blood vessels were observed invading the germinal center (black arrow).

**Figure 2:**
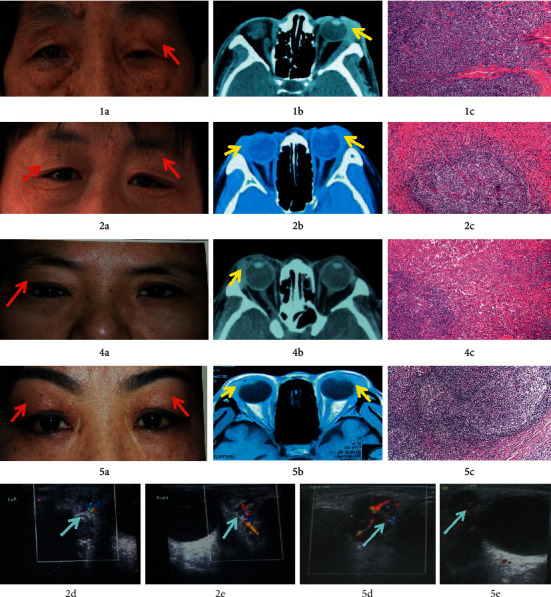
Case 1: 1(a) ∼ 1(c), case 2: 2(a) ∼ 2(e), case 4: 4(a) ∼ 4(c), and case 5: 5(a) ∼ 5(e). Preoperative appearance of the eyes (red arrows in 1(a), 2(a), 4(a), and 5(a)). CT/MRI showed swollen masses in the lacrimal gland area (yellow arrows in 1(b), 2(b), 4(b), and 5(b)). Pathological images (1(c), 2(c), 4(c), and 5(c)). Color ultrasonography showed masses in the lacrimal gland area with abundant blood flow (blue arrows in 2(d), 2(e), 5(d), and 5(e)).

*Case* 3. A 76-year-old female was admitted to hospital as “right eye swelling for more than 2 months.” Previous topical hormone therapy showed no apparent effect. After admission, the color Doppler ultrasonography, orbital CT, and MRI were performed, and a space-occupying lesion was found in the lacrimal gland area of the right eye. Surgical resection was performed, and postoperative pathological examination showed lacrimal gland CD (Figure 1). Data of the other 4 patients are shown in Figure 2.

## 4. Discussion

CD is also known as giant lymph node hyperplasia, as well as the vascular follicular lymphatic hyperplasia [4]. Though CD is clinically rare, it can occur in any part of the lymphatic system. CD is less common in extranodal tissues, and the involvement of orbital tissue, that is, lack of lymphatic tissue, is rarer. Although the orbital tissue lacks lymphatic vessels and lymph nodes, lymphoid tissue hyperplasia can occur when the immune system is disturbed, resulting in corresponding pathological changes [5]. CD has also been reported to involve different parts of the orbital and eyeball. Liu et al. [6] reported 1 case of exudative retinal detachment with systemic symptoms involving the retinal choroid. Jiang et al. [7] reported a case involving the optic nerve, inferior rectus, and internal rectus, without systemic complications. Liu et al. [8] reported 2 cases of lacrimal gland involvement with space-occupying lesions in the lacrimal gland, accompanied by eyelid swelling and ptosis of the upper eyelid, which were similar to the clinical manifestations of the subjects in the study. Five patients with CD involving lacrimal glands were included in the study. Case 1 was accompanied by sublingual gland lobular enlargement, and the other 4 patients had no systemic manifestations. It is suggested that we should pay attention to the systemic lymphatic system and blood system for the lymphatic tissue proliferative disease with the initial occurrence in the eyes to avoid missed diagnosis.

The pathogenesis of CD has not been determined yet, and current studies have demonstrated mainly from HHV-8 virus infection and IL-6 immune regulation. It is speculated that the potential mechanism can be that the infected virus encodes IL-6 or related peptides, stimulates the proliferation of B cells and inhibits apoptosis, and at the same time upregulates the expression of VEGF and generates new blood vessels [9]. With human anti-IL-6 receptor antibodies to treat 7 cases of plasma cell type CD and mixed type CD, Nishimoto [10] found that fever and other symptoms disappeared, and the follicular hyperplasia was reduced pathologically, which confirmed the pathological and physiological significance of IL-6 in the pathogenesis of CD. However, only part of the phenomenon can be explained with the mechanism. Some scholars have also explored other mechanisms besides HHV-8 and IL-6, such as TNF, MCSF, IL-1 [11], IL-5 [12], CMV [13], and EBV [14, 15], and proposed that they might be related to the occurrence of CD. However, there is a lack of studies with large samples. HIV testing in the study showed negative results in all cases. Case 4 showed positive in CMV, HSV-1, and VZV, which belonged to the same herpesvirus family as HHV; as herpesviruses were clearly correlated with lymphoproliferative diseases [9], the patient was considered to be closely related to viral infection.

In terms of clinical manifestations, CD lacks specificity, and pathology is still required for a clear diagnosis. Liu and Chen [16] pointed out that only the hyaline-vascular type can be definitely diagnosed based on morphology, while the plasma cell type must be determined in the combination with clinical and pathological findings after excluding other diagnoses. As for lymphoproliferative diseases in the lacrimal gland area, inflammatory pseudotumor, IgG4-related eye disease, and orbital lymphoma have raised great challenges to the differential diagnosis of this disease [17]. Inatani et al. [18] reported a case of multicenter plasma cell CD with orbital inflammatory pseudotumor as the first symptom, which later developed into non-Hodgkin's lymphoma. Snead et al. [19] reported that, among 27 patients with orbital lymphoma who were completely cured after surgical resection, one patient was diagnosed as CD after repeated pathological examinations. Jing et al. [20, 21] also proposed that CD has a certain relationship with IgG 4 disease. In agreement with the abovementioned views, the author also believes that CD, IgG 4-related eye disease, and orbital inflammatory pseudotumor have some connection and even transformation. Case 5 in this study was diagnosed as IgG 4-related eye disease with repeated examinations after the second recurrence, which also gives support to this point of view. The main distinguishing points should be combined with clinical manifestations and pathological findings. The clinically rare CD should be included in the differential diagnosis of an asymptomatic and painless orbital mass that is indicated as lymphatic proliferative disease pathologically. As for the diagnosis of IgG 4-related eye diseases, Japanese scholars have proposed an internationally recognized standard in 2014, suggesting that IgG 4-related eye diseases can be definitely diagnosed when combining imaging, pathological, and hematological findings [22]. Studies have shown that 36% of patients with orbital inflammatory pseudotumor are IgG 4-related eye diseases, and they respond quickly and repeatedly to glucocorticoid therapy, while some patients with CD respond poorly to glucocorticoid therapy [23]. Case 3 in this study was insensitive to preoperative glucocorticoid therapy. The pathological diagnosis of CD can be determined after ruling out other possibilities, and the clear differentiation point is not available in the lack of a large amount of data, which needs further in-depth study.

Imaging examination is a powerful tool in the diagnosis of orbital diseases. Zeng Qiao *et al.* proposed that orbital CD imaging was short of specificity and the soft tissue mass of the localized CD was homogeneous with equal density, without invasion to the bone, while the diffuse lesion usually involved bilateral lacrimal glands. For the enhancement pattern, the plasma cell type was continuous, while the hyaline-vascular type was progressive [5]. In this study, 1 of the 3 patients with unilateral invasion was of a hyaline-vascular type, and 1 of the 2 patients with bilateral lacrimal gland invasion was of the plasma cell type. Color Doppler ultrasonography showed abundant blood flow signals in all the cases, which were not completely consistent with the image characteristics described by previous scholars. It may be correlated to the lack of experience in imaging diagnosis of ocular CD, which is insufficient to describe the characteristics comprehensively. When the pathology is clear, the imaging examination is of great significance in localization diagnosis but has no obvious advantages in qualitative diagnosis.

Histopathologically, CD can be divided into three types: the hyaline-vascular type, plasma cell type, and mixed type. Clinically, there are localized lesions and diffuse lesions [24]. The combination of clinical and histopathological features is beneficial to guide the treatment and predict the prognosis of patients. Surgical resection of localized CD can always achieve a curative effect. Jones et al. [25] treated a 17-year-old female patient with localized CD by surgical resection, and the operation received good effects and showed no recurrence for 10 months. Kang et al. [26] performed a surgical resection on a 54-year-old male patient with locally orbital CD without recurrence for 13 months. For diffuse CD, the clinical manifestations are invasive, especially the plasma cell type with systemic symptoms and the possibility of malignant transformation. No unified treatment scheme is available yet, and the combination of operation, chemotherapy, and radiotherapy is often required [27]. The same treatment principle is applied to CD that has invaded the eyes. Song and Jeong [28] reported 1 patient with systemic symptoms and invasion of the retinal choroid. With the CHOP chemotherapy (cyclophosphamide, doxorubicin, vincristine, and prednisolone) scheme, the fundus symptoms disappeared with a significant effect. Ide et al. [29] suggested that rituximab combined with glucocorticoid should be the preferred nonsurgical treatment for diffuse CD, but it has not been widely used in the ophthalmic clinical practice. During the close follow-up of Jiang Jiang et al. [7] for patients under orbital CD resection, no recurrence was noted. No recurrence was found after resection of the orbital lesion with postoperative radiotherapy by Jiang et al. [30] and after surgical resection with postoperative glucocorticoid therapy by Liu et al. [8]. In this study, all patients were treated with surgical resection of lesions, and the pathological characteristics showed that 3 patients were of the plasma cell type and 1 patient was of the hyaline-vascular type. They were given glucocorticoid therapy after operation, and it was suggested to be further treated in the oncology department, which was in line with the principle of CD diagnosis and treatment. In this study, Case 1 had no recurrence for 2 years after surgery but died due to a cardiovascular accident. Case 4 underwent surgical treatment 4 years ago and 1 year ago, showing CD of the plasma cell type pathologically. At present, the repeated surgery for the recurrence of the disease shows malignant transformation to the plasma cell tumor in pathology. The patient died 2 years after chemotherapy in the follow-up period. Combined with the literature, we can conclude that the hyaline-vascular type has a good prognosis, with a low recurrence rate and malignant transformation rate, while for patients of the plasma cell type and mixed type, attention should be paid to the occurrence of systemic tumors after surgery, they should be followed up closely, and the treatment in the combination of nonsurgical therapy should be conducted. The prognosis of patients with malignant transformation is poor.

In conclusion, Castleman's disease (CD) of the lacrimal gland is a rare orbital lymphoproliferative disease, lacking specificity in clinical manifestations and imaging examination. It is difficult to differentiate the disease from orbital inflammatory pseudotumor and orbital lymphoma, and its diagnosis still depends on pathological examination. The disease is mainly treated with surgical resection. When necessary, systemic chemoradiotherapy is combined for treatment. The prognosis is good in the absence of malignant transformation.

## Figures and Tables

**Table 1:** Clinical manifestations of 5 patients with CD of the lacrimal gland.

*Case*	1	2	3	4	5
*Gender*	*M*	*F*	*F*	*F*	*F*
*Age*	76	61	76	46	44
*Site*	*OS*	*OU*	*OD*	*OD*	*OU*
*Time*(*month*)	36	60	2	48	12
*Neoplasm*	+	+	+	+	+
*Boundary*	+	+	−	+	+
*Pain*	−	−	−	−	−
*Swelling/ptosis*	+	+	+	+	+
*Restriction of eye movement*	+	+	−	+	−
*Diplopia*	−	+	−	−	−
*Others*	*Sublingual gland lobular enlargement*			*CMV*(+)	
				*HSV-*1(+)	
				*VZV*(+)	
*Therapeutic regimen*	*Surgical resection*	*Surgical resection*	*Surgical resection*	*Surgical resection*	*Surgical resection*
*Pathological type*	*CD* (*plasma cell*)	*CD* (*hyaline-vascular*)	*CD* (*plasma cell*)	*CD* (*transformation to plasma cell tumor*)	*CD* (*plasma cell*)
*Prognosis*	*No recurrence*	*No recurrence*	*No recurrence*	*Recurrence*	*Recurrence*
	*Died due to cardiovascular accident*			*Chemotherapy*	*Progression to the IgG*4*-related eye disease*
				*Died due to a progressive disease*	
*Response to glucocorticoid*	+	+++	−	*No use*	+

**Table 2:** Pathological findings of 5 patients with CD of the lacrimal gland.

*Case*	1	2	3	4	5
*CD*79*a*	+		+		
*CD*20	+	+	+	+	+
*CD*3	+	+	+	+	+
*CD*5	+		+		
*CD*21	*FDC net*	*FDC net*	*FDC net*	*FDC net*	*FDC net*
*CD*23	*FDC net*		*FDC net*		
*CD*10	−	−	+ (*germinal center*)		
*CyLinD*1	−		−		
22*Bcl-*2	+ (*partially*)	+ (*outside germinal center*)	+ (*outside germinal center*)		+ (*outside germinal center*)
*Bcl-*6				+ (germinal center)	
*CD*31	+	+	+		
*CD*34		+			+
*CD*38		+	+	+	+
*CD*138		+	+	+	+
*CD*68				+ (*scattered*)	
*MUM-*1				+	
*Ki-*67	+ (*scattered*)	+ (*germinal center*)	+ (*germinal center*)	+	+ (*germinal center*)
*λ*	+	+	+	−	
*k*	+	+	+	+	

## Data Availability

The data used to support the findings of the study are available from the corresponding author upon request.
